# Cholesterol Efflux Capacity and Cardiovascular Disease: The Ludwigshafen Risk and Cardiovascular Health (LURIC) Study

**DOI:** 10.3390/biomedicines8110524

**Published:** 2020-11-21

**Authors:** Andreas Ritsch, Angela Duerr, Patrick Kahler, Monika Hunjadi, Tatjana Stojakovic, Guenther Silbernagel, Hubert Scharnagl, Marcus E. Kleber, Winfried März

**Affiliations:** 1 Department of Internal Medicine, Medical University of Innsbruck, 6020 Innsbruck, Austria; duerr.angela@web.de (A.D.); Patrick.kahler@tirol-kliniken.at (P.K.); monika.hunjadi@i-med.ac.at (M.H.); 2 Clinical Institute of Medical and Chemical Laboratory Diagnostics, University Hospital Graz, 8036 Graz, Austria; stojakovic@gmx.at; 3 Division of Angiology, Department of Internal Medicine, Medical University of Graz, 8036 Graz, Austria; guenther.silbernagel@yahoo.com; 4 Clinical Institute of Medical and Chemical Laboratory Diagnostics, Medical University of Graz, 8036 Graz, Austria; hubert.scharnagl@klinikum-graz.at (H.S.); winfried.maerz@synlab.com (W.M.); 5 Vth Department of Medicine, Medical Faculty Mannheim, Heidelberg University, 68167 Mannheim, Germany; marcus.kleber@medma.uni-heidelberg.de; 6 Synlab Academy, Synlab Holding Deutschland GmbH, 86156 Augsburg, Germany

**Keywords:** high-density lipoprotein, cholesterol efflux capacity, cardiovascular risk, mortality, dysfunctional HDL

## Abstract

(1) Background and Aims: Efforts to reduce coronary artery disease (CAD) by raising high-density lipoprotein (HDL) cholesterol (HDL-C) have not been uniformly successful. A more important factor than HDL-C may be cellular cholesterol efflux mediated by HDL, which has been shown to be associated with CAD. In this report, we analyzed the influence of cardiovascular biomarkers and risk factors on cholesterol efflux in a prospective observational study of patients referred to coronary angiography. (2) Methods: HDL-mediated efflux capacity was determined for 2468 participants of the Ludwigshafen Risk and Cardiovascular Health (LURIC) study who were referred to coronary angiography at baseline between 1997 and 2000. Median follow-up time was 9.9 years. Primary and secondary endpoints were cardiovascular and all-cause mortality, respectively. (3) Results: Cholesterol efflux strongly correlated with HDL-related markers including HDL cholesterol, HDL phospholipids, and apolipoproteins AI and AII, as well as HDL particle concentration, which was not seen for low density lipoprotein (LDL) markers including LDL cholesterol and apoB. Cholesterol efflux was associated negatively with C-reactive protein (CRP), fibrinogen, interleukin-6 (IL-6), and serum amyloid A. Cardiovascular mortality was higher in patients in the lowest cholesterol efflux quartile. This association was weakened, but not fully abolished, after adjustment for HDL cholesterol. (4) Conclusions: We demonstrate that cholesterol efflux was associated with HDL-composition as well as inflammatory burden in patients referred for coronary angiography, and that this inversely predicts cardiovascular mortality independently of HDL cholesterol.

## 1. Introduction

Low levels of high-density lipoprotein cholesterol (HDL-C) have been shown to represent an independent risk marker for atherosclerotic cardiovascular disease in the general population [1]. However, there is a growing body of evidence that raising HDL cholesterol levels may not consistently protect from atherosclerosis. Data from several cohorts have revealed a flattening, or even an increase, of the risk for coronary artery disease (CAD) at high concentrations of HDL cholesterol [2,3,4,5]. Patients with genetically elevated apoA-I and HDL-C are not at reduced risk for cardiovascular disease [6], although this was not evident in another study from the same research group [7,8]. Interventional studies targeting the elevation of HDL-C with cholesteryl ester transfer protein (CETP) inhibitors or niacin (on top of statins) have not provided evidence that the increased HDL levels were protective [2]. These observations point towards the presence of other actions of HDL that are not readily reflected by HDL-C. A key function of HDL among numerous actions is the ability to promote the efflux of cholesterol from peripheral cells and initiate the shuttling of cholesterol back to the liver [3,4,5]. The reverse cholesterol transport is considered atheroprotective by transferring excess cholesterol from the periphery back to the liver where it is secreted into the bile or converted into bile acids. Cholesterol efflux from cholesterol-laden macrophages represents an initial step within this pathway and has been shown to prevent atherosclerosis in animal models [4]. In the present study, we extend this observation to patients referred to coronary angiography. To obtain further insight into the regulation of cholesterol efflux we investigated the associations of cholesterol efflux capacity with cardiovascular mortality and a number of typical cardiovascular biomarkers and risk factors.

## 2. Experimental Section

### 2.1. Study Design and Participants

We studied 2468 participants of the LUdwigshafen RIsk and Cardiovascular Health (LURIC) study [6]. Inclusion criteria were: German ancestry, clinical stability except for acute coronary syndromes, and the availability of a coronary angiogram. The indications for angiography in individuals in clinically stable condition were chest pain and/or noninvasive test results consistent with myocardial ischemia. Individuals suffering from acute illness other than acute coronary syndromes, chronic non-cardiac diseases, or malignancy within the past 5 years and subjects unable to understand the purpose of the study were excluded. The study was approved by the Ethics Committee at the Aerztekammer Rheinland-Pfalz and was performed to conform with the declaration of Helsinki (837.255.97 (1394), approved 8 January 1999). Informed written consent was obtained from all participants. Reporting of the study conforms to STROBE along with references to STROBE and the broader EQUATOR guidelines [7]. CAD was assessed by angiography with maximum luminal narrowing estimated by visual analysis. As in previous studies, clinically relevant CAD was defined as the occurrence of ≥1 stenosis of 20% in ≥1 of 15 coronary segments. Individuals with stenoses <20% were considered not to have CAD; 52 (9.2%) and 396 (20.8%) cardiovascular deaths occurred during 9.9 years of follow-up in patients without or with coronary stenosis, respectively. For additional subgroup analysis, patients without a history of an acute event but at high risk of coronary artery disease were classified by absence of an acute event and having a pooled cohort equation level greater than 7.5 [8]. Diabetes mellitus was diagnosed when plasma glucose was >1.25 g/L in the fasting state or >2.00 g/L 2 h after an oral glucose load [9], or when antidiabetic medical treatment was prescribed. Hypertension was diagnosed when systolic and/or diastolic blood pressure exceeded 140 and/or 90 mm Hg, respectively, or when a patient was on antihypertensive medication. Data of cholesterol efflux capacity, plasma lipids, lipoproteins, and coronary angiograms were complete for all 2468 individuals included in statistical analyses.

Information on vital status was obtained from local registries. Of the 2468 individuals studied, 717 deaths (29.1%) occurred during a median follow-up of 9.9 (8.5–10.7) years. The follow-up for total mortality was complete, and cause of death could not be determined for 16 out of the 717 deceased. Cardiovascular death included sudden death, fatal myocardial infarction, death due to congestive heart failure, death immediately after intervention to treat CAD, fatal stroke, and other causes of death due to CAD.

### 2.2. Laboratory Procedures

For all analyses, fasting blood samples were collected prior to angiography. The standard laboratory methods have been described [6]. Triglycerides and cholesterol were quantified with enzymatic reagents from WAKO (Neuss, Germany) on a WAKO 30R analyzer. Lipoproteins were separated using a combined ultracentrifugation-precipitation method (β-quantification) in all LURIC participants: Plasma was ultra-centrifuged at a density of d = 1.0063 kg/L (30,000 rpm for 18 h). VLDL in the supernatant were removed. In the remainder, LDL (and IDL) were precipitated with phosphotungstic acid/MgCl_2_. Centrifugation (10,000 rpm for 5 min) was performed to separate precipitated LDL (and IDL) from HDL in the remainder. Concentrations of small (7–8.5 nm), medium (8.5–10 nm), and large (10–13 nm) HDL particles were analyzed with NMR spectroscopy (NUMARES AG, Regensburg, Germany) as described previously [10]. The total concentration of HDL particles was used to calculate relative contents of HDL components per particle. Biologically effective HDL cholesterol (HDL-Cˡ) was calculated using a recently developed formula based on measurements of HDL cholesterol and serum amyloid A (SAA) [11]:

(1) $$HDL-C^{\prime }=20.14\times {\left(0.213\times \ln\left(\ln\left(SAA\right)+1\right)+\left(0.073\times \ln\left(\ln\left(SAA\right)+1\right)-0.283\right)\sqrt{HDL-C}-0.176\right)}^{2}.$$

Cholesterol efflux capacity was quantified in blood samples as previously described [12]. All samples measured were from the same batch; storage time (at −70 °C) did not differ between all samples included in this study. Additionally, we tested whether cycles of freezing and thawing had an influence on our efflux measurements and found no indication that this was the case, as consistently reported by Rohatgi et al. [13].

Briefly, J774 cells derived from a murine macrophage cell line were plated in DMEM Hybri-Max (D2650, Sigm-Aldrich, St. Louis, MO, USA). with 1% fetal bovine serum and radiolabeled with 2 μCi of 3H cholesterol per milliliter. After labeling, the cells were washed 1× with PBS, equilibrated for 4 h with medium containing 0.2% BSA (essentially fatty-acid free) (A7030, Sigma-Aldrich, St. Louis, MO, USA). Cells were incubated with 0.3 mM cAMP (C3912, Sigma-Aldrich, St. Louis, MO, USA)) to upregulate ABCA1. Subsequently, efflux medium containing 2.8% apolipoprotein B–depleted serum prepared by precipitating apoB containing lipoproteins with a mix of 8.2% tungstophosphoric acid hydrate (1.00583.0100, Merck. Darmstadt, Germany) and 6.2% 1 M MgCl2 (2189.2, Roth, Karlsruhe, Germany) was added for 4 h. All steps were performed in the presence of 2 µg per milliliter acyl-coenzyme A cholesterol acyltransferase inhibitor (Sc-215839A, Santa-Cruz Biotechnology, Santa Cruz, CA, USA). Liquid scintillation counting was used to quantify the efflux of radioactive cholesterol from the cells. Percent efflux was calculated using the following formula: [(microcuries of 3H cholesterol in medium containing 2.8% apolipoprotein B-depleted serum − microcuries of 3H cholesterol in serum-free medium)/microcuries of 3H cholesterol in cells extracted before the efflux step] × 100. To correct for inter-assay variation across plates, a pooled serum control was included on each plate. Values for serum samples from patients were given as a percentage of this control (% C). CETP was determined with the use of an enzyme-linked immunosorbent assay employing a CETP-specific recombinant single-chain antibody as the coating antibody and an affinity-purified polyclonal anti-CETP antibody as the detection antibody, respectively [14,15]. Adiponectin serum levels were determined by ELISA (Biovendor Laboratory Medicine Inc., Brno, Czech Republic) according to the manufacturer’s instructions. ADMA and homocysteine measurements were performed by reversed-phase HPLC [16]. Interleukin-6 was measured using a high sensitivity enzyme immunoassay (R&D Systems, Wiesbaden, Germany). High sensitivity C-reactive protein concentrations and serum amyloid A was determined by immunonephelometry (Dade Behring, Marburg, Germany). Fibrinogen was measured using the Clauss method (Stago Diagnostica/Roche Mannheim, Germany). All assays were performed in triplicate.

Briefly, J774 cells derived from a murine macrophage cell line were plated in DMEM Hybri-Max (D2650, Sigm-Aldrich, St. Louis, MO, US). with 1% fetal bovine serum and radiolabeled with 2 μCi of 3H cholesterol per milliliter. After labeling, the cells were washed 1× with PBS, equilibrated for 4 h with medium containing 0.2% BSA (essentially fatty-acid free) (A7030, Sigma-Aldrich, St. Louis, MO, USA). Cells were incubated with 0.3 mM cAMP (C3912, Sigma-Aldrich, St. Louis, MO, USA)) to upregulate ABCA1. Subsequently, efflux medium containing 2.8% apolipoprotein B–depleted serum prepared by precipitating apoB containing lipoproteins with a mix of 8.2% tungstophosphoric acid hydrate (1.00583.0100, Merck. Darmstadt, Germany) and 6.2% 1 M MgCl2 (2189.2, Roth, Karlsruhe, Germany) was added for 4 h. All steps were performed in the presence of 2 µg per milliliter acyl-coenzyme A cholesterol acyltransferase inhibitor (Sc-215839A, Santa-Cruz Biotechnology, Santa Cruz, CA, US). Liquid scintillation counting was used to quantify the efflux of radioactive cholesterol from the cells. Percent efflux was calculated using the following formula: [(microcuries of 3H cholesterol in medium containing 2.8% apolipoprotein B-depleted serum − microcuries of 3H cholesterol in serum-free medium)/microcuries of 3H cholesterol in cells extracted before the efflux step] × 100. To correct for inter-assay variation across plates, a pooled serum control was included on each plate. Values for serum samples from patients were given as a percentage of this control (% C). CETP was determined with the use of an enzyme-linked immunosorbent assay employing a CETP-specific recombinant single-chain antibody as the coating antibody and an affinity-purified polyclonal anti-CETP antibody as the detection antibody, respectively [14,15]. Adiponectin serum levels were determined by ELISA (Biovendor Laboratory Medicine Inc., Brno, Czech Republic) according to the manufacturer’s instructions. ADMA and homocysteine measurements were performed by reversed-phase HPLC [16]. Interleukin-6 was measured using a high sensitivity enzyme immunoassay (R&D Systems, Wiesbaden, Germany). High sensitivity C-reactive protein concentrations and serum amyloid A was determined by immunonephelometry (Dade Behring, Marburg, Germany). Fibrinogen was measured using the Clauss method (Stago Diagnostica/Roche Mannheim, Germany). All assays were performed in triplicate.

### 2.3. Statistical Analysis

Data normally distributed are presented as mean ± SD. CETP, triglycerides, adiponectin, interleukin-6 (IL-6), and C-reactive protein (CRP) exhibited a skewed distribution and are presented as median and interquartile (Q1, Q3) range. Parameters not normally distributed were transformed logarithmically for statistical analyses. The effects of cardiovascular risk factors, CAD status, and intake of lipid-lowering drugs on cholesterol efflux levels were determined using general linear models, with cholesterol efflux as the dependent variable and age, intake of statins, CAD status, diabetes mellitus, smoking history (never, former, or current), LDL cholesterol, HDL cholesterol, and triglycerides as independent variables. Multivariable adjustment was performed for baseline levels of age, intake of statins, CAD status (none, stable CAD, unstable CAD, non-ST-elevation myocardial infarction (NSTEMI), or ST-elevation myocardial infarction (STEMI)), smoking status, LDL cholesterol, HDL cholesterol, triglycerides, and metabolic syndrome/type 2 diabetes mellitus. All statistical tests were two-sided; *p* < 0.05 was considered significant. The SPSS 22.0 statistical package (SPSS Inc., Chicago, IL, USA) was used. The hazard ratio plots were drawn using R statistical software v3.5.3 (http://www.r-project.org) and the ‘rms’ package v5.1-3.

## 3. Results

### 3.1. Study Participants

Serum samples from 2468 individuals from the LURIC study were available for measurement of cholesterol efflux capacity. Clinical and biochemical characteristics of the study population are presented in Table 1, classified into quartiles of cholesterol efflux capacity. No differences were observed for age, waist to hip ratio, frequency of diabetes mellitus, and smoking.

Participants within the highest efflux quartile showed slightly increased baseline levels of systolic blood pressure, lower BMIs, and lower rates of male sex and patients receiving lipid-lowering therapy. In the same group of patients, levels of total cholesterol, HDL cholesterol, apoAI, and apoAII were increased with no effects seen for LDL cholesterol and apoB. The rate of patients without CAD was highest in high-cholesterol efflux patients.

During a median follow-up time of 9.9 years, 717 participants (29.1%) died, of which 62.5% (*n* = 448) of all deaths were caused by cardiovascular events.

### 3.2. Cholesterol Efflux Capacity and Cardiovascular Risk Factors

**Figure 1:**
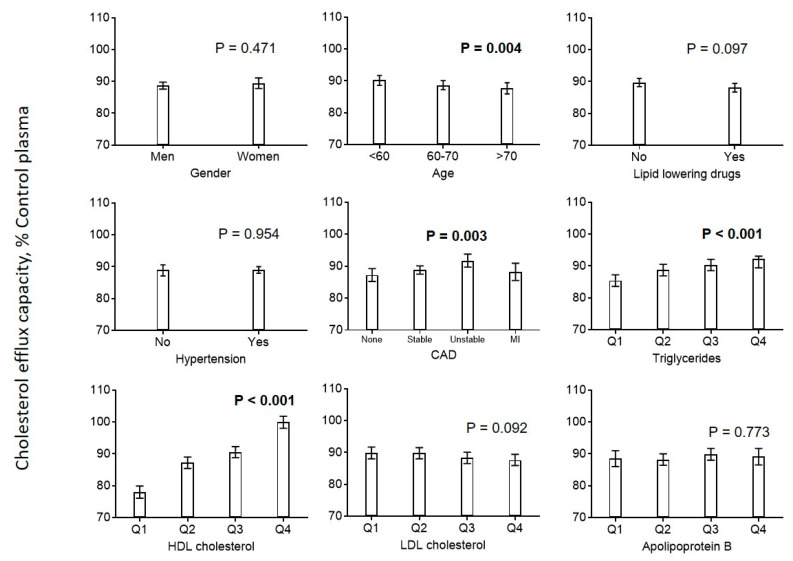
Association of cardiovascular risk factors with cholesterol efflux. Diagrams show the estimated marginal means with 95% confidence intervals obtained from a general linear model adjusted for use of statins, age, CAD, diabetes mellitus, smoking, LDL cholesterol, HDL cholesterol, and triglycerides. Age, LDL cholesterol, HDL cholesterol, and triglycerides (log-transformed) were included as continuous rather than categorical covariables. *p* values are given for comparison with the first category of each variable. *p* values < 0.05 are shown in bold. CAD = coronary artery disease.

Next, we investigated the potential associations among lifestyle and clinical factors, lipoprotein parameters, and cholesterol efflux capacity. We found an elevated estimated cholesterol efflux capacity in younger patients, patients with higher triglyceride levels, and patients with unstable CAD. We found a strong association with HDL-C, but no association with LDL-C or apoB (Figure 1).

**Figure 2:**
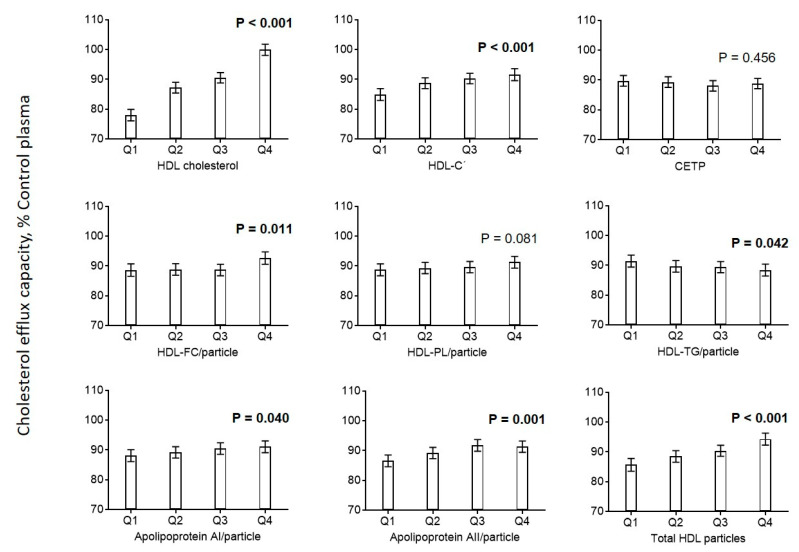
Association of HDL parameters with cholesterol efflux. Diagrams show estimated marginal means with 95% confidence intervals obtained from a general linear model adjusted for use of statins, age, CAD, diabetes mellitus, smoking, LDL cholesterol, HDL cholesterol, and triglycerides. Age, LDL cholesterol, HDL cholesterol, and triglycerides (log-transformed) were included as continuous rather than categorical covariables. *p* values are given for comparison with the first category of each variable. *p* values < 0.05 are shown in bold. Biologically effective HDL cholesterol (HDL-Cˡ) was calculated using a recently developed formula based on measurements of HDL cholesterol and serum amyloid A (SAA) [11]. HDL-Cˡ = effective high-density lipoprotein cholesterol.

Looking at the related parameters of HDL in greater detail, we found positive associations between cholesterol efflux capacity and effective HDL, HDL-unesterified cholesterol, apoA-I, and apoA-II, and a negative association with HDL triglycerides (Figure 2). The distribution of cholesterol efflux capacity measurements according to cardiovascular risk factors and HDL parameters is shown in Supplemental Materials Figures S4 and S5.

Cholesterol efflux correlated strongly with NMR-derived particle concentrations of all HDL size classes, with the highest correlation observed for large HDL particles (Figure 2 and Supplemental Materials Figure S1, left panel). When we provisionally used relative concentrations of different HDL size classes, the correlation of small HDLs with cholesterol efflux turned negative, while the correlations of efflux with intermediate and large ones stayed positive (Supplemental Materials Figure S1, right panel).

### 3.3. Markers of Inflammation

**Figure 3:**
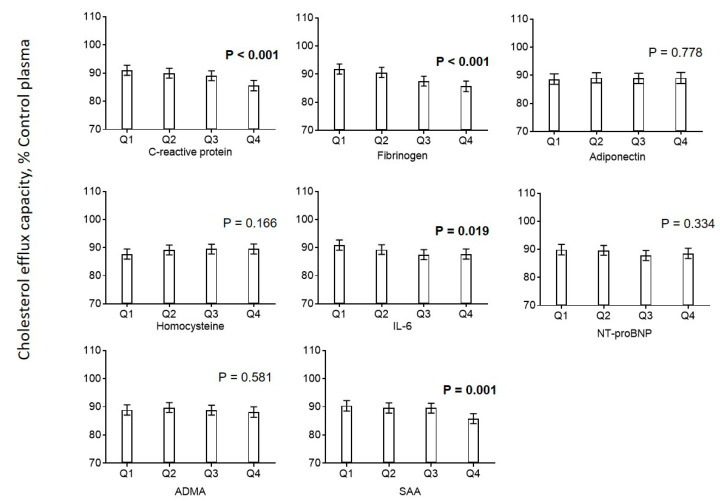
Association of markers of inflammation with cholesterol efflux. Diagrams show estimated marginal means with 95% confidence intervals obtained from a general linear model adjusted for use of statins, age, CAD, diabetes mellitus, smoking, LDL cholesterol, HDL cholesterol, and triglycerides. Age, LDL cholesterol, HDL cholesterol, and triglycerides (log-transformed) were included as continuous rather than categorical covariables. *p* values are given for comparison with the first category of each variable. *p* values < 0.05 are shown in bold

Efflux capacity was negatively associated with CRP, fibrinogen, IL-6 and serum amyloid A (SAA) (Figure 3). No associations were found for homocysteine, NT-proBNP, and ADMA. Additionally, we found that biologically effective HDL cholesterol (HDL-Cˡ) calculated using a recently developed formula based on measurements of HDL-C and serum amyloid A was positively associated with cholesterol efflux capacity (Figure 2) [11].

### 3.4. Cardiovascular Mortality

**Figure 4:**
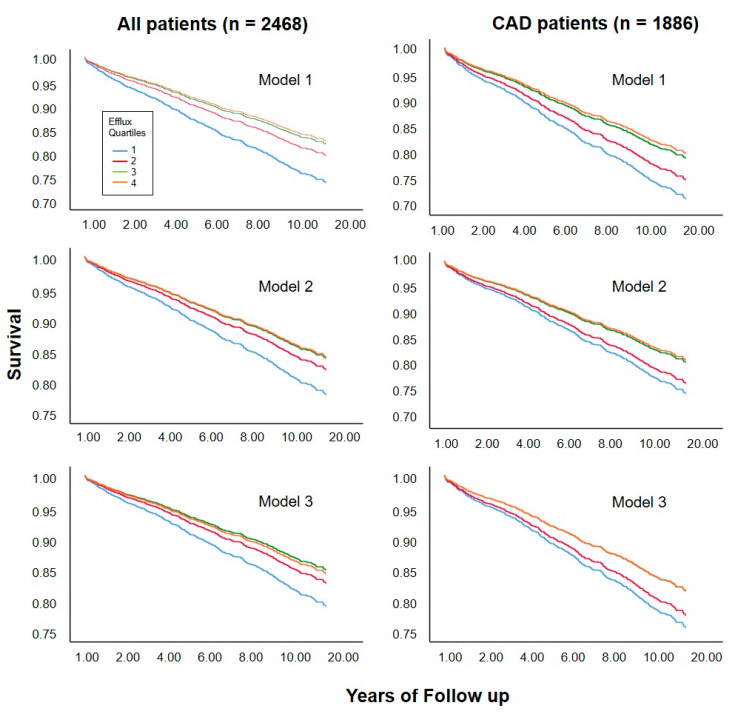
Survival curves according to quartile of cholesterol efflux capacity. Curves were estimated in proportional-hazards models without adjustment (model 1), adjusted for age and sex (model 2), or for age, sex, and traditional risk factors for cardiovascular disease including use of statins, CAD, diabetes mellitus, smoking, triglycerides, and LDL cholesterol (model 3).

Of the 2468 individuals studied, 717 deaths (29.1%) occurred during a median follow-up of 9.9 years. We found a clear association between cholesterol efflux capacity and cardiovascular mortality (Table 2, model 1) in the whole study population as well as in a subgroup analysis of 1886 subjects with angiographically verified CAD status at baseline. This association remained significant after including an adjustment for age and sex (model 2), as well as after an additional adjustment for known risk markers like use of statins, CAD, diabetes mellitus, smoking, triglycerides, and LDL cholesterol (model 3). We found similar effects concerning the association between cholesterol efflux capacity and cardiovascular death in a subgroup analysis of patients without cardiovascular events but with a high risk of coronary artery disease based on the pooled cohort equation [8]. This association remained significant even after full adjustments (Supplementary Materials Table S1). In the whole study population, additional adjustment for HDL-C weakened but did not fully abolish the association between cholesterol efflux capacity and cardiovascular mortality (model 4). This was not true for further adjustments, including additional HDL related parameters (model 5) or markers of inflammation (model 6). The same picture was seen in additional calculations considering cholesterol efflux capacity as a continuous variable, as shown by spline curves in Supplementary Materials Figures S2 and S3. Corresponding survival curves according to quartiles of cholesterol efflux capacity are shown in Figure 4.

## 4. Discussion

In recent years, several clinical studies have investigated the relationship between cholesterol efflux capacity and cardiovascular risk. A recent meta-analysis of 20 articles showed that increased high-density lipoprotein cholesterol efflux capacity was associated with a lower risk of cardiovascular disease [17]. Authors stated that the heterogeneity and evidence of publication bias highlight the need for larger prospective studies. In good agreement with this meta-analysis we found an inverse association between cholesterol efflux capacity and mortality in participants of the Ludwigshafen Risk and Cardiovascular Health (LURIC) study, and this remained significant even after correcting for traditional risk factors for cardiovascular disease [18]. Considering cholesterol efflux capacity as a major functional property of HDL, our data suggest that HDL function is associated with cardiovascular risk by means that are not directly associated with HDL-C level and other traditional risk factors for cardiovascular disease. However, once we included either HDL cholesterol or apo AI into our logistic regression models, HDL-mediated cholesterol efflux was no longer associated with cardiovascular or total mortality. This was due to the high correlations between these measurements, which means that there was no predictive information beyond the traditional cardiovascular risk factors. We therefore did not specifically analyze integrated discrimination or net classification improvement in our study.

In this prospective observational study of patients referred to coronary angiography, we further analyzed the influence of cardiovascular biomarkers and risk factors on cholesterol efflux. Expanding beyond previous work, we investigated a number of parameters influencing cholesterol efflux capacity, including HDL composition, and size, along with subclinical inflammation. We therefore provide important new data herein regarding the influence of cardiovascular risk factors on cholesterol efflux capacity. Accordingly, cholesterol efflux was correlated with lipid and lipoprotein parameters, with the strongest associations seen with HDL-C, apolipoprotein AI and AII, and the concentration of large HDL particles. We did not find an association with LDL cholesterol or plasma apolipoprotein B, but efflux was negatively associated with plasma triglycerides. Looking at the composition of HDL particles, we found a positive association between cholesterol efflux and HDL-free cholesterol, but no association with HDL phospholipids and, interestingly, a negative association with HDL triglycerides.

Our results indicate that in addition to the most frequently investigated HDL-C, various parameters including lipids, proteins, and even size might play an important role in HDL function in reverse cholesterol transport. Overall, the association between diverse HDL parameters with cholesterol efflux capacity is a strong indication that the assay used in this study mainly reflected HDL-mediated cholesterol efflux capacity. Recently, we showed that SAA might modify the biological functions of HDL in several clinical settings [11]. As expected, the newly introduced parameter “effective” HDL based on measurements of HDL cholesterol and SAA was strongly associated with mortality in our study population. We found a strong association between effective HDL (HDL-Cˡ) and cholesterol efflux in the present study, although this was, however, slightly exceeded by HDL-C itself. In addition, we found a negative association between SAA plasma concentration and cholesterol efflux capacity. This is in good agreement with several reports showing a negative influence of SAA on HDL function in terms of cholesterol efflux and reverse cholesterol transport [19,20].

Cholesterol efflux is the first major step within the reverse cholesterol transport, and the technology used has been shown to be useful in several clinical studies by us as well as by others [12,13,18]. It is, however, a limitation of our study that measurement of cholesterol efflux capacity does not reflect reverse cholesterol transport completely. Thus, even although we observed that cholesterol efflux capacity was related to the concentrations of HDL and its subclasses, our ex vivo model did not permit any conclusions in regard to the contributions of the diverse subcellular mechanisms of cholesterol mobilization, including ABCA1 and SR-BI in vivo. Another limitation of our study may be that we included only patients referred to coronary angiography. Therefore, our findings cannot be transferred to the general population.

The participants of the LURIC study were recruited between 1997 and 2001, then followed for fatal events. Lipoprotein parameters and cholesterol efflux capacity were measured once at baseline, but we were not able to adjust for possible moderate fluctuations of these parameters during the follow-up. We also have no information on long-term changes of medication or the initiation of other preventive measures during the follow-up period that may have impacted our results. However, we believe that any such changes would not have produced a major bias. Our data are in good agreement with recent data of a secondary prevention study suggesting that cholesterol efflux capacity is a useful prognostic and therapeutic surrogate for secondary prevention of CAD [21].

The major strengths of this work are the detailed clinical and metabolic characterization of the LURIC participants and the long duration of the follow-up with a large number of fatal cardiovascular events.

In summary, our study supports the concept of dysfunctional HDL. In contrast to previous results reporting on the association between HDL-C and cardiovascular mortality, data from the present study show that cholesterol efflux was associated with cardiovascular mortality both in patients with and without verified CAD. Additionally, cholesterol efflux capacity was shown to be associated not only with various HDL parameters, but also with markers of inflammation. Our data, therefore, demonstrate that cholesterol efflux capacity constitutes a risk factor for cardiovascular mortality, and might reflect HDL functions in reverse cholesterol transport.

## Figures and Tables

**Table 1:** Clinical and biochemical characteristics of study participants at baseline according to cholesterol efflux capacity quartiles.

Efflux (% C, Median, Range)	All (n = 2468)	Quartile 1 60.4 (21.8–73.7) (n = 617)	Quartile 2 80.9 (73.7–87.9) (n = 617)	Quartile 3 95.1 (87.9–102.6) (n = 617)	Quartile 4 119.1 (102.7–178.0) (n = 617)	*p* ^a^
Age (years, mean ± SD)	62.8 ± 10.4	63.4 ± 10.6	62.7 ± 10.6	62.6 ± 10.2	62.8 ± 10.2	0.279
Male sex (%)	68.1	73.4	68.2	70.7	60.1	<0.001
Body mass index (kg/m^2^, mean ± SD)	27.5 ± 4.1	27.8 ± 4.1	27.8 ± 4.2	27.5 ± 4.0	27.0 ± 4.1	0.005
Waist hip ratio (mean ± SD)	0.96	0.96 ± 0.07	0.96 ± 0.08	0.96 ± 0.08	0.95 ± 0.08	0.068
Total cholesterol (mg/dL, mean ± SD)	208 ± 44	203 ± 46	205 ± 43	210 ± 43	215 ± 45	<0.001
LDL^c^ cholesterol (mg/dL, mean ± SD)	116 ± 35	114 ± 35	115 ± 35	117 ± 35	118 ± 36	0.429
HDL^c^ cholesterol (mg/dL, mean ± SD)	39 ± 11	35 ± 10	38 ± 9	40 ± 11	44 ± 11	<0.001
Effective HDL (mg/dL, mean ± SD)	35 ± 18	30 ± 17	33 ± 16	36 ± 17	41 ± 19	<0.001
Apolipoprotein AI (mg/dL, mean ± SD)	130 ± 25	121 ± 24	127 ± 23	132 ± 24	141 ± 26	<0.001
Apolipoprotein AII (mg/dL, mean ± SD)	42 ± 10	39 ± 10	41 ± 9	42 ± 9	45 ± 10	<0.001
Apolipoprotein B (mg/dL, mean ± SD)	104 ± 25	104 ± 25	104 ± 25	105 ± 25	104 ± 25	0.944
Triglycerides (mg/dL, median, Q1 to Q3)	143 (106–198)	145 (104–202)	140 (106–200)	147 (110–200)	139 (104–193)	0.346
Systolic blood pressure (mmHg, mean ± SD)	141	139 ± 23	142 ± 24	141 ± 23	142 ± 24	0.015
Diastolic blood pressure (mmHg, mean ± SD)	81	80 ± 12	82 ± 12	81 ± 11	81 ± 11	0.097
CETP ^c^ (μg/mL, median, Q1 to Q3)	1.12 (0.86–1.49)	1.12 (0.86–1.47)	1.09 (0.84–1.46)	1.14 (0.87–1.56)	1.15 (0.87–1.50)	0.152 ^b^
Diabetes mellitus (%)	28.9	31.0	32.9	33.7	29.0	0.286
Lipid lowering therapy (%)	49.7	52.7	49.4	51.9	44.9	0.029
CAD ^c^ None (%)	23.0	20.9	21.6	23.5	25.9	
Stable CAD (%)	46.6	46.8	49.3	45.1	45.2	
Unstable CAD (troponin T−) (%)	18.9	18.0	15.6	20.6	21.4	
NSTEMI ^c^ or STEMI (troponin T+) (%)	11.5	14.3	13.6	10.9	7.5	0.001
Smoking						
Never (%)	37.5	34.0	36.8	37.6	41.5	
Past (%)	43.2	45.7	43.8	42.6	40.8	
Current (%)	19.3	20.3	19.4	19.8	17.7	0.262

^a^ Analysis of variance or logistic regression, respectively, adjusted for age and sex. ^b^ ANOVA of logarithmically transformed values. ^c^ LDL, low density cholesterol; HDL, high density lipoprotein; CETP, cholesteryl ester transfer protein; CAD, coronary artery disease; NSTEMI, non-ST-segment elevation myocardial infarction.

**Table 2:** Hazard ratio for cardiovascular death according to cholesterol efflux.

All Patients (*n* = 2468)						
**Efflux Quartile**	**Model 1 HR (95% CI)**	***p***	**Model 2 HR (95% CI)**	***p***	**Model 3 HR (95% CI)**	***p***
**1st (*n* = 617)**	1.0^reference^		1.0^reference f^		1.0^reference^	
**2nd (*n* = 617)**	0.756 (0.590–0.968)	0.026	0.800 (0.625–1.025)	0.077	0.804 (0.628–1.031)	0.086
**3rd (*n* = 617)**	0.658 (0.509–0.852)	0.001	0.711 (0.549–0.920)	0.010	0.698 (0.539–0.905)	0.007
**4th (*n* = 617)**	0.632 (0.488–0.818)	0.001	0.701 (0.540–0.910)	0.008	0.726 (0.559–0.943)	0.016
	**Model 4 HR (95% CI)**	***p***	**Model 5 HR (95% CI)**	***p***	**Model 6 HR (95% CI)**	***p***
**1st (*n* = 617)**	1.0^reference^		1.0^reference^		1.0^reference^	
**2nd (*n* = 617)**	0.841 (0.655–1.080)	0.174	0.863 (0.671–1.109)	0.249	0.911 (0.705–1.177)	0.475
**3rd (*n* = 617)**	0.755 (0.579–0.984)	0.037	0.786 (0.602–1.024)	0.075	0.798 (0.607–1.049)	0.105
**4th (*n* = 617)**	0.815 (0.620–1.070)	0.141	0.851 (0.648–1.120)	0.250	0.850 (0.641–1.125)	0.256
**CAD Patients (*n* = 1886)**						
	**Model 1 HR (95% CI)**	***p***	**Model 2 HR (95% CI)**	***p***	**Model 3 HR (95% CI)**	***p***
**1st (*n* = 483)**	1.0^reference^		1.0^reference^		1.0^reference^	
**2nd (*n* = 482)**	0.854 (0.659–1.106)	0.232	0.907 (0.700–1.176)	0.461	0.904 (0.697–1.172)	0.445
**3rd (*n* = 465)**	0.695 (0.527–0.917)	0.010	0.732 (0.555–0.966)	0.027	0.731 (0.554–0.965)	0.027
**4th (*n* = 456)**	0.6665 (0.503–0.881)	0.004	0.716 (0.540–0.949)	0.020	0.731 (0.551–0.970)	0.030
**Efflux Quartile**	**Model 4 HR (95% CI)**	***p***	**Model 5 HR (95% CI)**	***p***	**Model 6 HR (95% CI)**	***p***
**1st (*n* = 483)**	1.0^reference^		1.0^reference^		1.0^reference^	
**2nd (*n* = 482)**	0.937 (0.721–1.219)	0.630	0.956 (0.735–1.244)	0.738	1.019 (0.778–1.335)	0.892
**3rd (*n* = 465)**	0.772 (0.582–1.025)	0.073	0.605 (0.602–1.067)	0.131	0.821 (0.613–1.100)	0.186
**4th (*n* = 456)**	0.799 (0.595–1.074)	0.137	0.620 (0.648–1.123)	0.233	0.838 (0.618–1.137)	0.257

Model 1: not adjusted. Model 2: adjusted for age and gender. Model 3: adjusted for age, gender, use of statins, CAD, diabetes mellitus, smoking, triglycerides, and LDL cholesterol. Model 4: adjusted for age, gender, use of statins, CAD, diabetes mellitus, smoking, triglycerides, LDL cholesterol, and HDL cholesterol. Model 5: adjusted for age, gender, use of statins, CAD, diabetes mellitus, smoking, triglycerides, LDL cholesterol, HDL cholesterol, apolipoprotein AI, apolipoprotein AII, and HDL-C’. Model 6: adjusted for age, gender, use of statins, CAD, diabetes mellitus, smoking, triglycerides, LDL cholesterol, HDL cholesterol, adiponectin, fibrinogen, and C-reactive protein (CRP). CI = confidence interval, HR = hazard ratio.

## Supplementary Materials

The following are available online https://www.mdpi.com/2227-9059/8/11/524/s1. Figure S1: Association of absolute and relative HDL particle concentration with cholesterol efflux; Figure S2: Spline curves showing hazard ratios for cardiovascular death according to cholesterol efflux capacity in the whole study population (*n* = 2468); Figure S3: Spline curves showing hazard ratios for cardiovascular death according to cholesterol efflux capacity in CAD patients (*n* = 1886); Figure S4: Box plots showing the distribution of cholesterol efflux capacity according to cardiovascular risk factors; Figure S5: Box plots showing the distribution of cholesterol efflux capacity according to HDL parameters; Table S1. Hazard ratio for cardiovascular death according to cholesterol efflux in 833 patients of the LURIC study without a history of an acute coronary event but with a high risk for CAD (pooled cohort equation > 7.5).
