# Supplementary material for: Cholesterol Efflux Capacity and Cardiovascular Disease: The Ludwigshafen Risk and Cardiovascular Health (LURIC) Study
Source: Biomedicines. 2020 Nov 21;8(11):524. doi: 10.3390/biomedicines8110524 (PMC7700479; doi:10.3390/biomedicines8110524)
Supplement: Supplementary file 1 [file biomedicines-08-00524-s001.pdf]

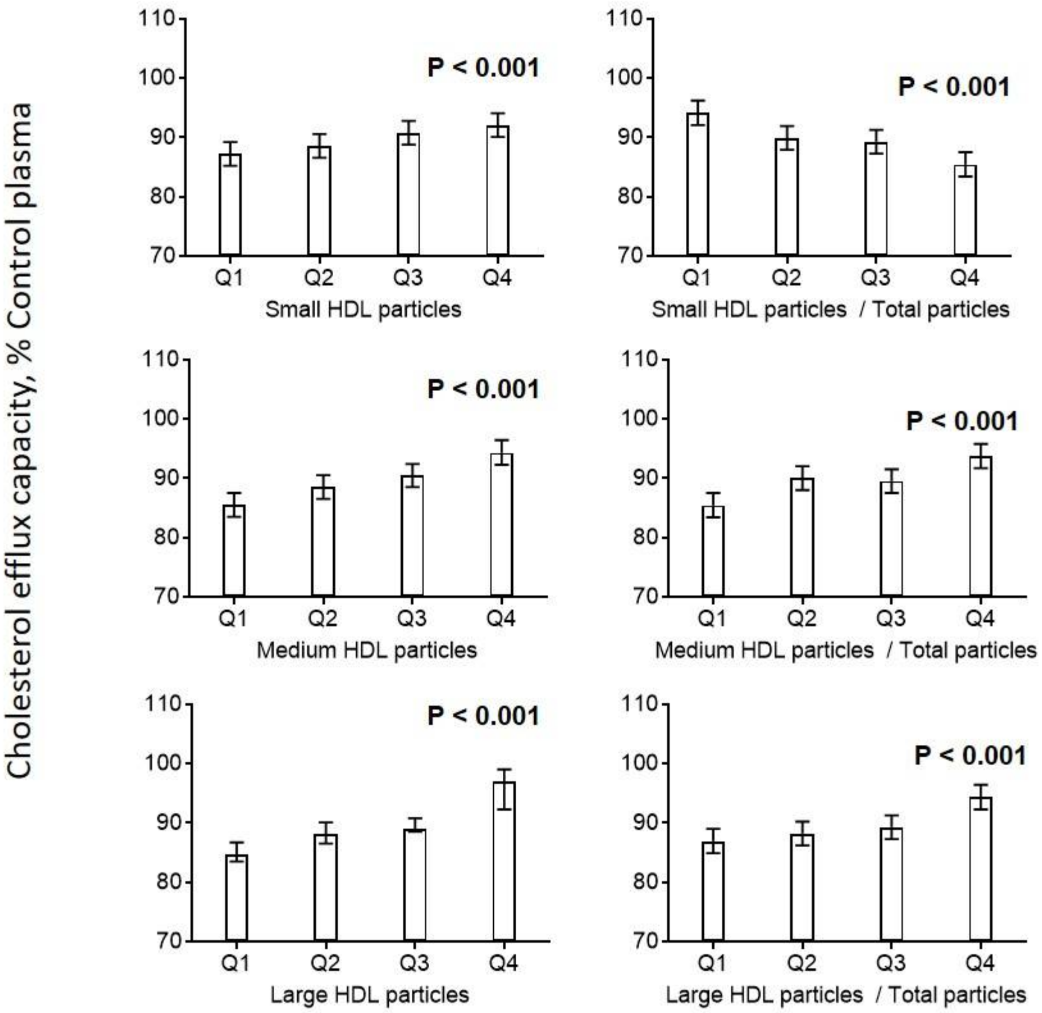

Supplemental figure 1. Association of absolute (left panel) and relative (right panel) HDL particle. concentration with cholesterol efflux. Diagrams are showing estimated marginal means and 95% confidence intervals obtained in a general; linear model, adjusted for the use of statins, age, CAD, diabetes mellitus, smoking, LDL cholesterol, HDL cholesterol and triglycerides. Age, LDL-cholesterol, HDL-cholesterol and triglycerides (log transformed) were included as continuous rather than categorical covariables. p values are given for comparison with the first category of each variable.

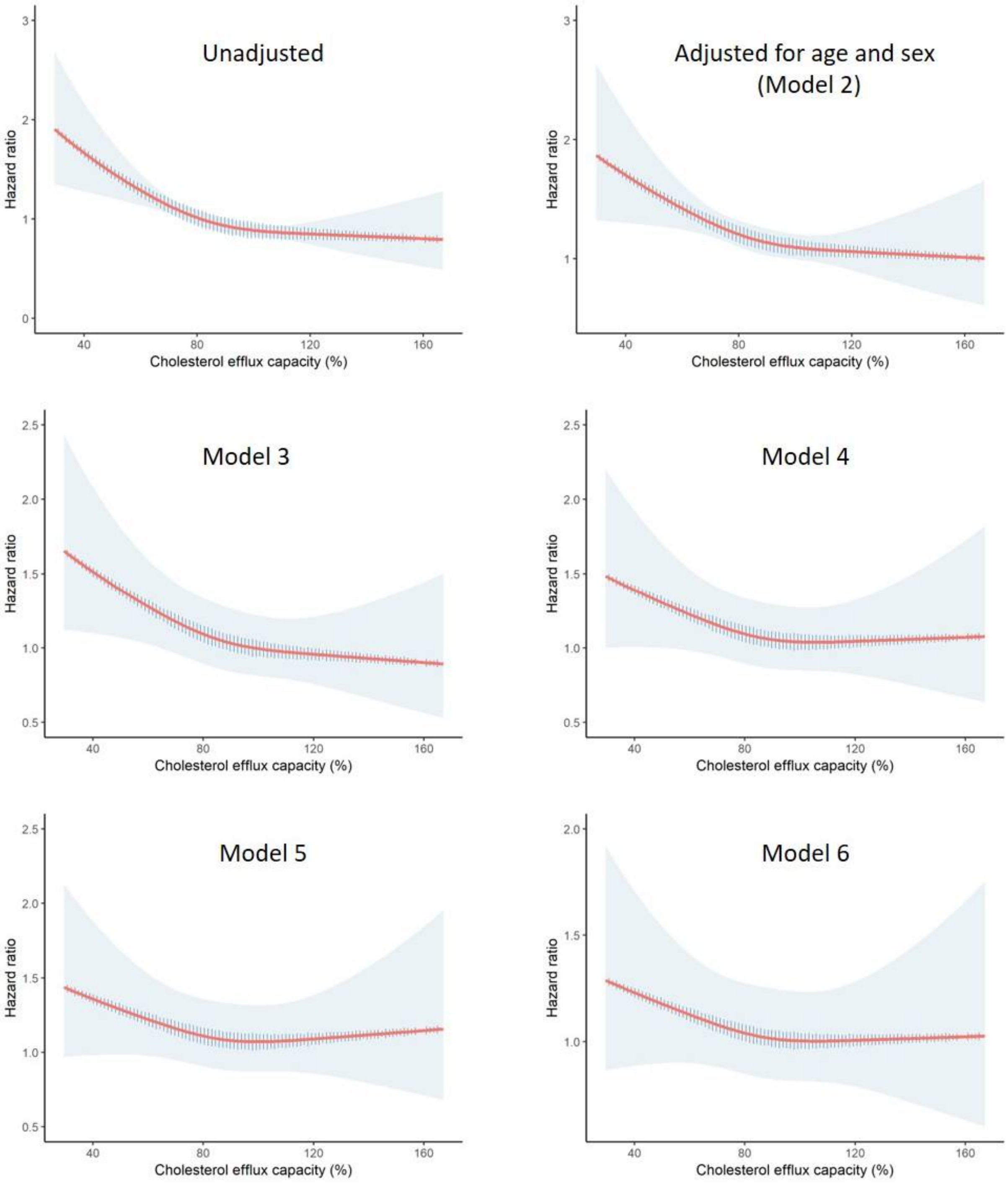

Supplemental figure 2. Spline curves showing hazard ratios for cardiovascular death according to cholesterol efflux capacity in the whole study population (n = 2468). Calculations were repeated with following models of adjustment: Model 1: not adjusted. Model 2: adjusted for age and gender. Model 3: adjusted for age, gender, use of statins, CAD, diabetes mellitus, smoking, triglycerides and LDL-cholesterol. Model 4: adjusted for age, gender, use of statins, CAD, diabetes mellitus, smoking, triglycerides, LDLcholesterol and HDL-cholesterol. Model 5: adjusted for age, gender, use of statins, CAD, diabetes mellitus, smoking, triglycerides, LDLcholesterol, HDL-cholesterol, apolipoprotein AI, apolipoprotein AII, HDL-C'. Model 6: adjusted for age, gender, use of statins, CAD, diabetes mellitus, smoking, triglycerides, LDLcholesterol, HDL-cholesterol, adiponectin, fibrinogen, C-reactive protein.

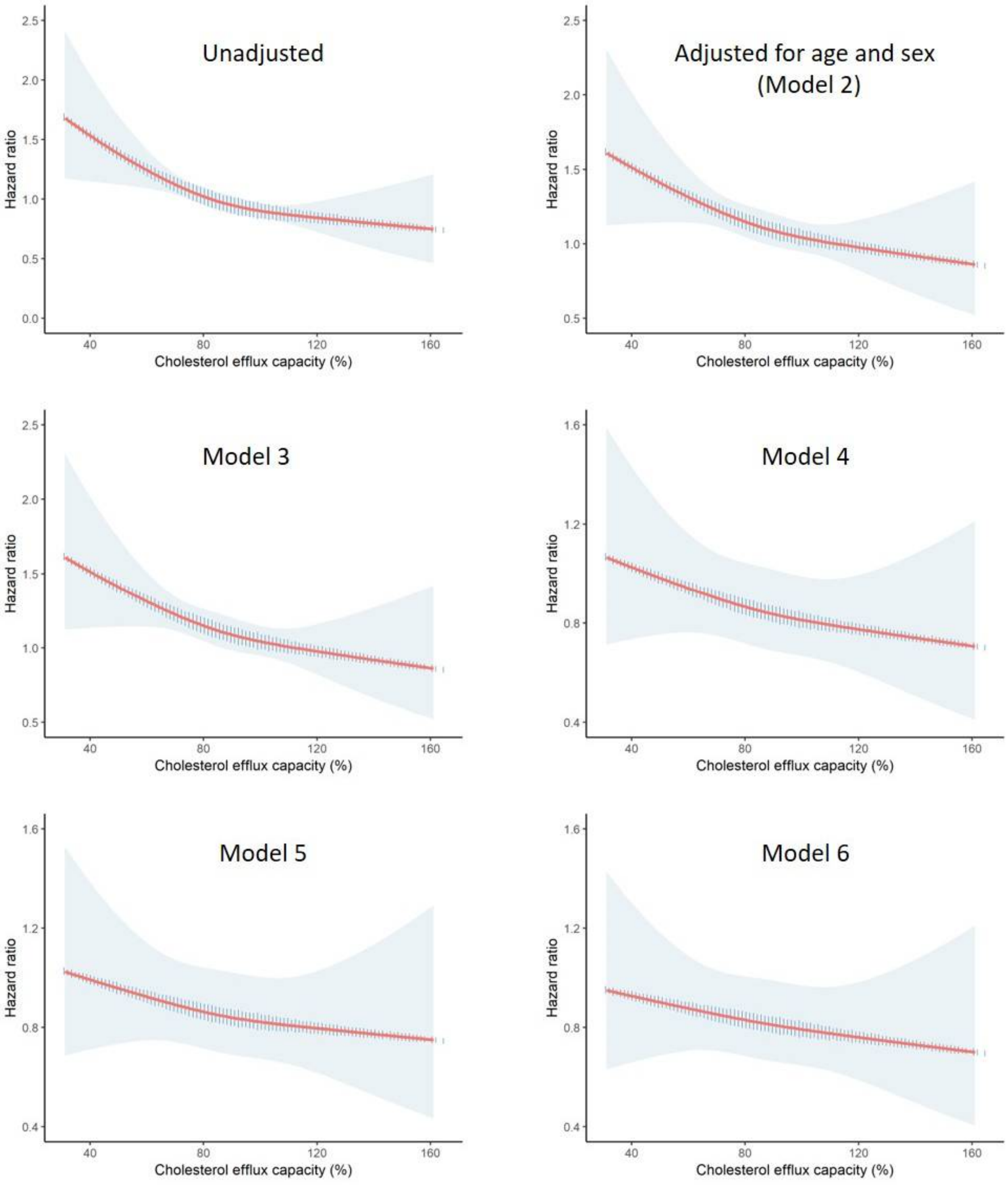

Supplementary figure 3. Spline curves showing hazard ratios for cardiovascular death according to cholesterol efflux capacity in CAD patients (n = 1886). Calculations were repeated with following models of adjustment: Model 1: not adjusted. Model 2: adjusted for age and gender. Model 3: adjusted for age, gender, use of statins, CAD, diabetes mellitus, smoking, triglycerides and LDL-cholesterol. Model 4: adjusted for age, gender, use of statins, CAD, diabetes mellitus, smoking, triglycerides, LDLcholesterol and HDL-cholesterol. Model 5: adjusted for age, gender, use of statins, CAD, diabetes mellitus, smoking, triglycerides, LDLcholesterol, HDL-cholesterol, apolipoprotein AI, apolipoprotein AII, HDL-C'. Model 6: adjusted for age, gender, use of statins, CAD, diabetes mellitus, smoking, triglycerides, LDLcholesterol, HDL-cholesterol, adiponectin, fibrinogen, C-reactive protein.

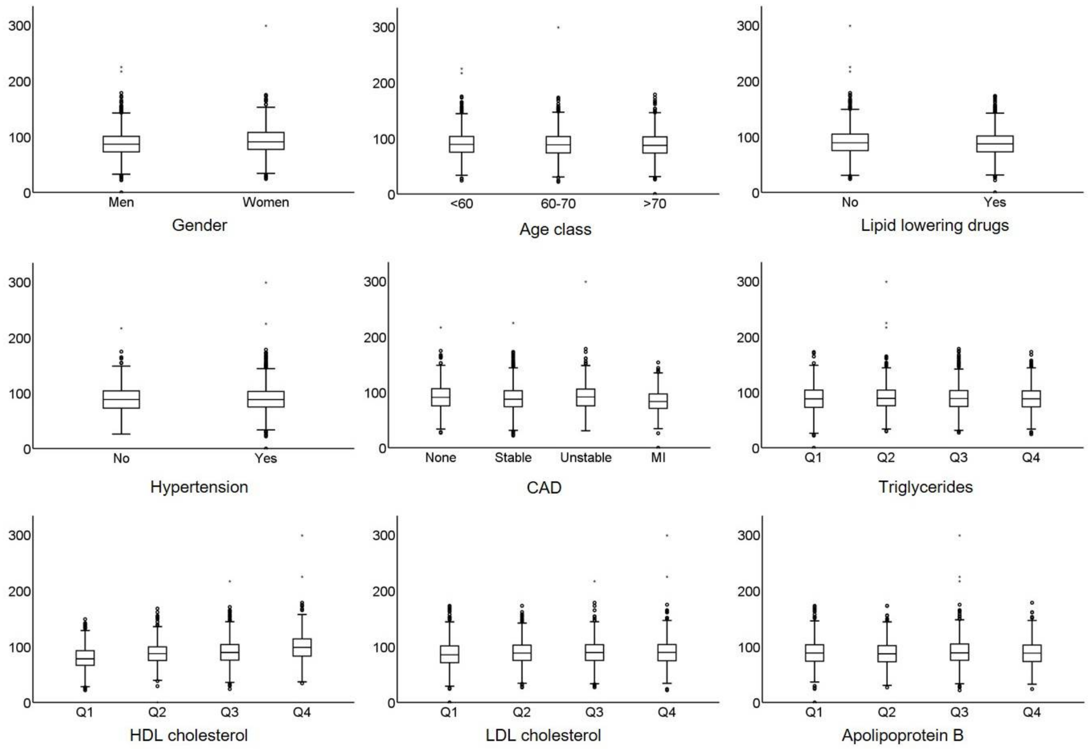

Supplemental figure 4. Box plots showing the distribution of cholesterol efflux capacity according to cardiovascular risk factors. Boxplots are displaying minimum, first quartile, median, third quartile, and maximum, as well as outliers.

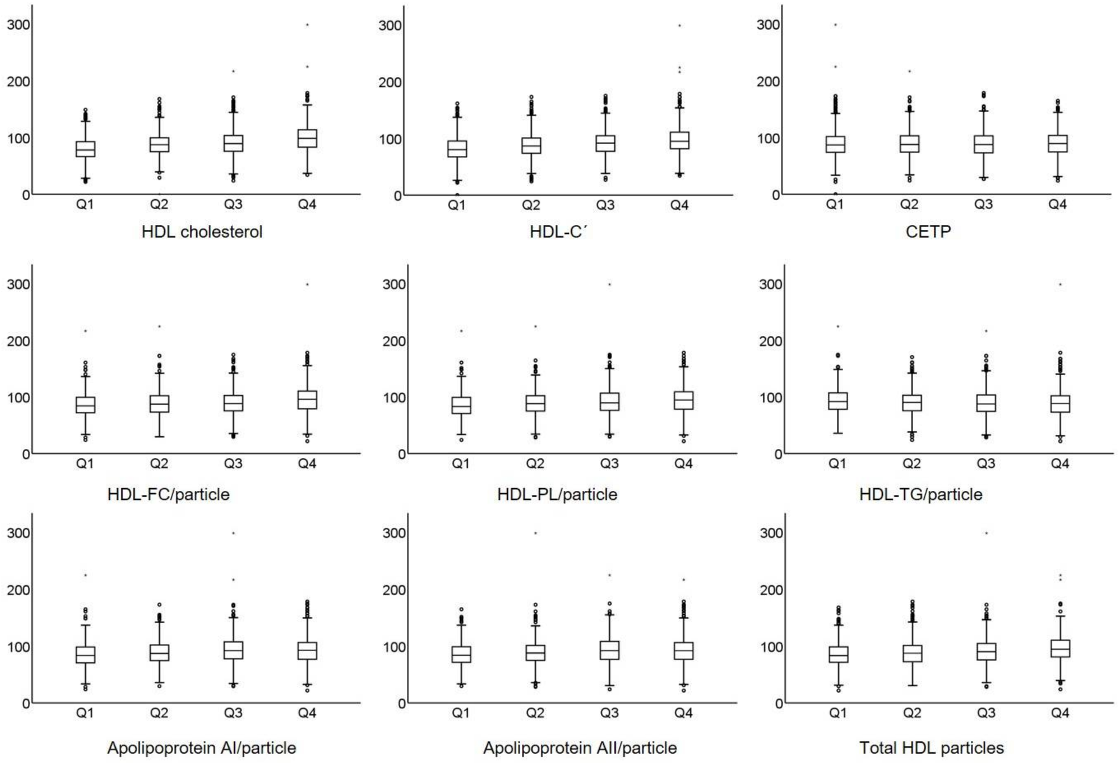

Supplemental figure 5. Box plots showing the distribution of cholesterol efflux capacity according to HDL parameters. Boxplots are displaying minimum, first quartile, median, third quartile, and maximum, as well as outliers.

Supplementary Table 1. Hazard ratio for cardiovascular death according to cholesterol efflux in 833 patients of the LURIC study without a history of an acute coronary event but with a high risk for CAD (pooled cohort equation > 7.5).

**Efflux Quartile**	**Model 1 HR (95% CI)**	**p**	**Model 2 HR (95% CI)**	**p**	**Model 3 HR (95% CI)**	**p**
1st (n=188)	1.0 reference		1.0 reference		1.0 reference	
2nd (n=216)	0.521 (0.324-0.863)	0.007	0.522 (0.325-0.840)	0.007	0.481 (0.298-0.774)	0.003
3rd (n=199)	0.500 (0.304-0.822)	0.006	0.500 (0.304-0.823)	0.006	0.484 (0.294-0.799)	0.005
4th (n=230)	0.547 (0.346-0.866)	0.010	0.550 (0.345-0.878)	0.012	0.533 (0.333-0.852)	0.009
	Model 4 HR (95% CI)	p	Model 5 HR (95% CI)	p	Model 6 HR (95% CI)	p
1st (n=188)	1.0 reference		1.0 reference		1.0 reference	
2nd (n=216)	0.514 (0.318-0.830)	0.006	0.537 (0.331-0.870)	0.012	0.558 (0.337-0.925)	0.024
3rd (n=199)	0.540 (0.326-0.897)	0.017	0.562 (0.337-0.935)	0.027	0.695 (0.412-1.174)	0.174
4th (n=230)	0.636 (0.405-1.086)	0.103	0.734 (0.443-1.217)	0.231	0.799 (0.476-1.342)	0.396

Model 1: not adjusted. Model 2: adjusted for age and gender. Model 3: adjusted for age, gender, use of statins, CAD, diabetes mellitus, smoking, triglycerides and LDL-cholesterol. Model 4: adjusted for age, gender, use of statins, CAD, diabetes mellitus, smoking, triglycerides, LDL-cholesterol and HDL-cholesterol. Model 5: adjusted for age, gender, use of statins, CAD, diabetes mellitus, smoking, triglycerides, LDL-cholesterol, HDL-cholesterol, apolipoprotein AI, apolipoprotein AII, HDL-C'. Model 6: adjusted for age, gender, use of statins, CAD, diabetes mellitus, smoking, triglycerides, LDL-cholesterol, HDL-cholesterol, adiponectin, fibrinogen, C-reactive protein. CI = confidence interval, HR = hazard ratio.
